# Usability Testing of a Digitized Interventional Prehabilitation Tool for Health Care Professionals and Patients Before Major Surgeries: Formative and Summative Evaluation

**DOI:** 10.2196/59513

**Published:** 2024-12-11

**Authors:** Andreas A Schnitzbauer, Charlotte Detemble, Sara Fatima Faqar-Uz-Zaman, Julia Dreilich, Lisa Mohr, Svenja Sliwinski, Dora Zmuc, Mark Siller, Johannes Fleckenstein

**Affiliations:** 1 Department of General, Visceral, Transplant and Thoracic Surgery Frankfurt University Hospital Goethe University Frankfurt Frankfurt Germany; 2 Institute of Sports Medicine Goethe University Frankfurt/Main Frankfurt Germany; 3 Faculty for Applied Informatics Hochschule Heilbronn Heilbronn Germany; 4 Department of Pain Medicine Hospital Landsberg am Lech Landsberg am Lech Germany

**Keywords:** usability testing, prehabilitation, MARS, Mobile Application Rating Scale, trustworthiness, surgical research, usability, prerehabilitation tool, tool, medical device, surgery, device, application, design, engineering, development

## Abstract

**Background:**

The development of a medical device requires strict adherence to regulatory processes. Prehabilitation in this context is a new area in surgery that trains, coaches, and advises patients in mental well-being, nutrition, and physical activity. As staff is permanently drained from clinical care, remote and digital solutions with real-time assessments of data, including patient-related outcome reporting, may simplify preparation before major surgeries.

**Objective:**

This study aimed to evaluate the usability engineering process for the Prehab App, a newly developed medical device, in order to identify and adapt any design and usability flaws found.

**Methods:**

We hypothesized that formative and summative usability testing would achieve 80% interrater and intrarater reliability and consistency and that the safety-relevant scenarios would uncover undetected risks of the medical device (stand-alone software class IIa). In total, 8 experts and 8 laypersons (patients and potential patients) were asked to evaluate paper-based mockups, followed by an evaluation of the minimal viable product (MVP) of the Prehab App at least more than 8 weeks later after instruction and training. The experts had to face 5 and the laypersons 6 usability scenarios. Their evaluations were measured with the Mobile App Rating Scale (MARS) and trustworthiness checklists (range 0-64, with higher scores indicating trustworthiness), and the usability scenarios were evaluated with the After Scenario Questionnaire (ASQ) and a judgment by an observer. The time taken for the scenarios was also recorded.

**Results:**

MARS achieved constant scores of more than 4 out of 5 points for both experts and laypersons. The mean trustworthiness score was 51.3 (SD 2.7) for the experts and 50.8 (SD 2.1) for the laypersons (*P*=.68) in task I. The interrater correlation, shown by the Fleiss-Kappa value, was 0.87 (range 0.85-0.89) for all raters (N=16), 0.86 (range 0.82-0.91) for the experts (n=8, 50%), and 0.88 (range 0.84-0.93) for the laypersons (n=8, 50%), reflecting almost perfect agreement between the raters. This indicated the high quality of the usability. The usability scenarios were performed with ease, except for the onboarding part, when the wearable was required to be connected; this took a considerable amount of time and was recognized as a challenge to good usability.

**Conclusions:**

The formative and summative evaluation of the Prehab App design resulted in good-to-acceptable results of the design and usability of the critical and safety-relevant areas of the medical device and stand-alone software. Usability testing improves medical devices early in the design and development process, reduces errors, and mitigates risks, and in this study, it delivered a profound ethical and medical justification for a randomized controlled trial (RCT) of the Prehab App in a remote setting as a next step in the development process.

**Trial Registration:**

German Registry for Clinical Trials (DRKS00026985); https://drks.de/search/en/trial/DRKS00026985

## Introduction

Prehabilitation is a new field in clinical medicine that focuses on the optimal preparation of a patient for an operation [1]. The concept is based on multiple pillars of short-term lifestyle modifications predominantly reflecting the World Health Organization’s (WHO) recommendations for physical activity and the European code against cancer [2,3]. Specifically, it focuses on physical activity, mental well-being, and optimal nutrition. With an evidence-based implementation of high-end prehab programs, complication rates can be reduced by up to 50% in major thoracic and abdominal surgical procedures [4-6]. This leads to a cost-saving effect of 30% [7,8] and has the potential to relieve payers by US $35 billion per year by otherwise increasing patient numbers by 5%-10% every year, which reflects an increase in revenues by US $20-$35 billion per year in Europe and North America.

Significant roadblocks to a broad and successful implementation into routine clinical treatment pathways are the high demand for resources, such as staff (physiotherapy, dedicated qualified nurses and doctors, space and equipment, etc.), patient compliance often necessitating repeated presurgical traveling to the hospitals, and, if applied remotely, a lack of real-time documentation of the exercises applied. The COVID-19 pandemic accelerated the development of digital solutions, which, however, are regulated rigorously by authorities as most app-based solutions require clearance by authorities and certification as a medical device. The certification process is complicated and requires verification and validation of the medical device, as well as usability testing of mockups, minimal viable products (MVPs), and prototypes, before being applied in a real patient setting among many other requirements outlined in the regulatory documents of the European Union (EU) and the Food and Drug Administration (FDA). This is to mitigate the potential risks identified in risk management during the development phase.

Usability testing thus needs to be set up to identify potential user errors that lead to hazardous situations for the patient. Here, we presented the stepwise usability testing of a newly developed medical device, the Prehab App, to identify and adapt its design and usability flaws before testing it in a randomized controlled trial (RCT) in the intended use in a remote setting and to obtain conformity with the regulatory requirements of the European Medical Device Regulation (MDR). The effects of successful usability testing are reduced time for the development of a medical device, simplification of training of end users, lower requirements for customer support, better compliance, and real-life penetration of the solution in its intended use and clinical setting. We hypothesized that an interrater correlation and internal consistency of more than 80% in a patient and an expert group was achievable in a stringent usability approach to the medical device’s high-risk scenarios.

## Methods

### Prework

The Prehab App has been developed in a stepwise approach following the MDR. The software development plan, requirements specification, and architecture were set up following the regulatory requirements of the MDR. The software was designed iteratively and included 3 design reviews and specific verification and validation procedures. Usability testing included team-based formative evaluations with group discussions and cognitive walk-throughs following the Deutsches Institut fur Normung (DIN; German industry norm) 62366:2016-05. This led to the setup of a summative evaluation with specific use scenarios derived from dedicated risk management following DIN ISO 14971:2019 (where ISO refers to the International Organization for Standardization).

### Specific Design Aspects of the Prehab App

Starting with a peer and patient survey in 2021, it became clear that a safety-by-design approach focusing on usability, patient safety, intuitive use, and the implementation of tools for structured assessment of the stand-alone software would be the key requirements and key success factors while designing it [9]. This required a clear overview of elements in the app, including clear pathways and an order of data entry fields allowing the necessary entries to generate a clearly defined and algorithmic output.

This was the key to the interface with the doctors: we used easy-to-assess risk data that did not require any additional support tools and that allowed the workflow to be easily implemented into daily practice [1,10-12]. We designed buttons in a way that a decision had to be made and that the decision was clear for the user. For example, if a date or a year was required, only a year or a specific date could be chosen from a scroll menu. In case a numeric input was necessary, the software would prohibit any entry except for numbers. This was developed in an iterative way using a “look and feel” principle.

**Figure 1:**
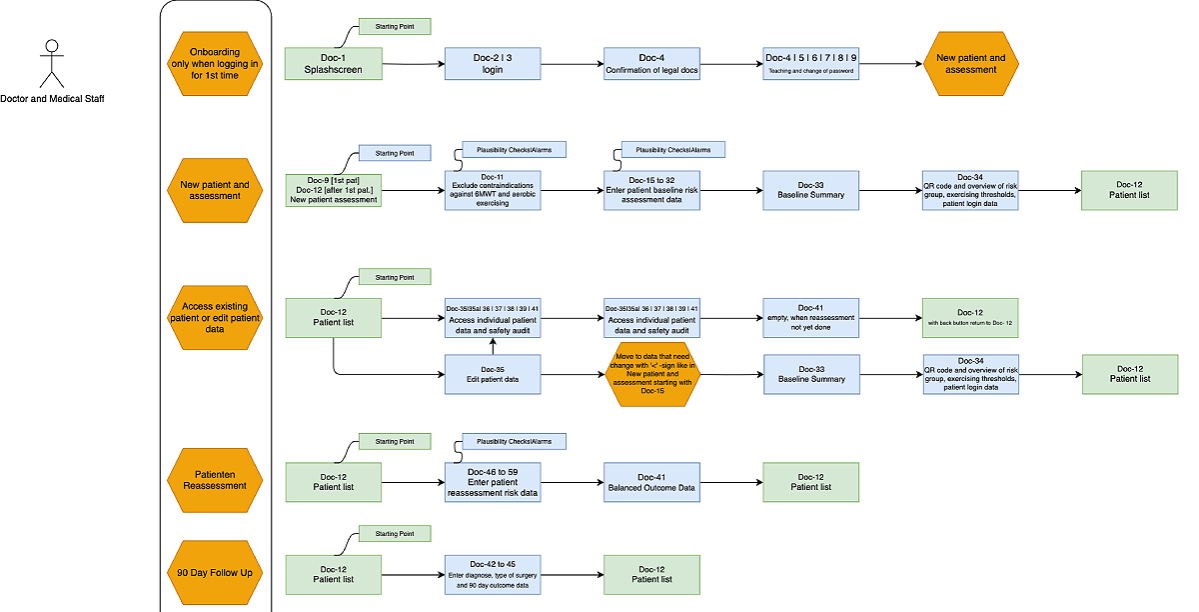
Screen workflows for the doctors' app. The workflow explains how the users get from one screen to the next, what the task and assessment will be, and where they will be prompted to go at the end of the assessment. The workflow is a logic baseline information for the onboarding of users and for the technically interested to dive deeper into the logic screen flow of a digital solution.

**Figure 2:**
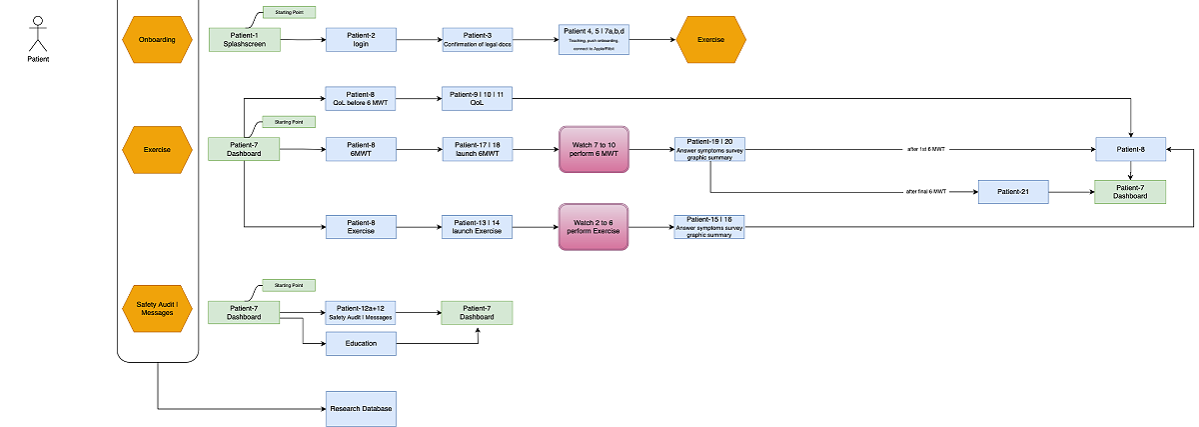
Screen workflows for the patients’ app. The workflow explains how the users get from one screen to the next, what the task and assessment will be, and where they will be prompted to go at the end of the assessment. The workflow is a logic baseline information for the onboarding of users and for the technically interested to dive deeper into the logic screen flow of a digital solution.

In the laypersons’ interface, the development, research, and design team followed the same approach, thinking from a patient’s perspective and the “keep it smart and simple” principle [9]. As outlined before, we conducted structured interviews with patients and potential patients to find out their requirements for a digital solution. This study was the final evaluation of usability in patients before systematic use in a remote setting in a clinical trial. In both apps, it was important to have a clear way from A to B and back to a dashboard area as the home screen that allowed reproducibility, repetition, and launch of new activities. After the design was finished, different safety-relevant scenarios were identified by risk management, and the scenarios were designed based on those definitions and findings. Three design reviews were the basis for the setup of the scenarios before launching the clinical investigation. Figure 1 and 2 show the resulting screen workflow in the Prehab App.

The version used in the clinical trial for usability testing was Prehab App version 1.0.0. The protocol has been published previously [11]. The results of the screening part of the trial with the risk assessment in the doctor’s app was recently published and showed good predictive potential of our risk assessment with 90-day postoperative outcomes [10]. The data on pulse assessment and accuracy in comparison with a certified electrocardiogram (ECG) are currently under review and available as open source. The data indicate a low mean absolute percentage error of <5% for the Apple iOS and Google Wear OS–based software solutions programmed within the Prehab App when compared with a certified ECG [12].

### Ethical Considerations

The Protego Maxima trial followed the MDR, DIN ISO 14155, and the Medizinproduktedurchführungsgesetz (MPDG; medical devices implementation law). The trial received a positive vote by the Frankfurt University Institutional Review Board (IRB) on November 11, 2021 (2021-483-MDR), and was approved by the Bundesinstitut für Arzneimittel und Medizinprodukte (BfArM) on February 7, 2022 (German Medical Devices Information and Database System [DMIDS]: 00013655; European Database on Medical Devices [EUDMAD]: CIV-21-07-037311; German Study Registry: DRKS00026985).

Notably, the informed consent obtained from the subjects studied allows publication of the data obtained from this trial in an aggregated and anonymized form, as presented here. In the case of future publications from secondary analysis by third parties, the sponsor will decide upon request how the data will be provided in an anonymous fashion. There was no compensation for subjects voluntarily participating in the trial.

### Subjects, Tests, and Procedures

Patients were approached in the outpatient clinic of our hospital. Doctors were randomly approached, focusing on a mix of residents and consultants, and were balanced for gender and age. Digital experts were located in the digital transformation office. The testers were divided into 2 groups: experts and laypersons. There were 3 different aspects of usability testing: a paper-based mockup evaluation, followed by an MVP evaluation (>8 weeks later) performed to identify design flaws in the Prehab App using the Mobile Application Rating Scale (MARS) [13] and the Trustworthiness Checklist [14,15], and aspects of the Aktionsbündnis für Patientensicherheit (APS; alliance for patient safety) checklist for digital apps [16]. The testers received a briefing using the patient/doctor information sheet of the software, including specific background information about the developer, the design, data safety and protection principles, and regulatory requirements followed in the development of the medical device, before they answered the questionnaires. The training material is provided in Multimedia Appendix 1.

Usability testing was derived from risk management for user groups. The testers were provided with a description of the task, including a short description, the precondition, and the expected condition after the task was finalized. Evaluators were provided with a regular flow. General workflows within the Prehab App were designed as a clear workflow without multiple variations to fulfill “safety by design” aspects. After each scenario, the testers answered the After Scenario Questionnaire (ASQ; scale from 1=strongly agree to 7=strongly disagree), and they evaluated their task performance as “done with ease,” “done with problems,” or “failed.” Additionally, the time to finalize each task was measured. Scenarios for the experts were for onboarding and entering the first patient (scenario D1), correcting a wrong data entry (scenario D2), onboarding the patient and explaining the app workflow (scenario D3), reading and clicking a safety audit (scenario D4), and performing a 90-day follow-up assessment (scenario D5). Laypersons had to perform onboarding (scenario P1), explore the app by navigating the tabs (scenario P2), start the 6-Minute Walk Test (6MWT) and an exercise (scenario P3), fill out the symptom checker after the exercise (scenario P4), and react to the notifications in the safety audit generated by the software (scenario P5).

Finally, usability testing of alarms during controlled exercising on a treadmill for 75 patients in task IIIb was performed (scenario P6). The alarms were set up in a way that the patients received vibration alarms, and pulse values displayed switched from green to red if they trained outside their predefined heart rate thresholds (too high or too low). The duration until they realized the alarms and adapted their behavior was recorded [17].

The usability protocols are provided in Multimedia Appendix 1.

### Scoring of the Questionnaires and Meaning of Scores

MARS was used to introduce a second standardized method that has proven to be of high value for interrater and intrarater reliability in the literature designed for digital software solutions. MARS has been specifically designed to assess the engagement, functionality, aesthetics, and information provided in a digital solution. It uses a Likert scale from 1 to 5; the higher the score, the better the quality [13,18-20].

The trustworthiness checklist initially introduced by van Haasteren et al [14,15] was combined with the online checklist of the APS [16]. The survey consisted of 64 questions, and each positive answer was scored as 1, to sum up to a maximum score of 64 points. Notably, not all questions that were answered positively with yes were necessarily associated with a positive or beneficial aspect within the tested app. However, the higher the score, the better the results, and each question supported the detection of specific flaws in trustworthiness and regulatory requirements, as well as data protection issues.

Every user facing the usability scenarios had to answer the ASQ developed by Lewis in 1995, which uses a 7-point scale (1-7), with the lowest value reflecting the highest satisfaction. The 3 questions asked targeted overall satisfaction in completing the task, satisfaction with the time it took, and satisfaction with the support material and information that were provided to exhibit the task.

### Statistical Analysis and Sample Size

Following Jakob Nielsen [21], as few as 5 evaluators can detect more than 95% of design and usability flaws. As the primary aim was to detect critical features of the app workflow via ratings and use scenarios, we assumed a κ coefficient of 0.8 to denote substantial agreement between raters. Therefore, 8 raters were required in each evaluator group (group 1, experts; groups 2, laypersons comprising patients and potential patients without a medical background from all social classes), assuming a 1-sided α error of .05 and a power of 80% for both the dichotomous and 5-point scales. For usability scenarios, the findings were descriptively evaluated and considered in the risk and benefit assessment after the clinical investigation to understand and mitigate the risk to acceptable areas. Comparisons between groups were performed using *t* tests and assuming an α error of 5% being statistically significant and relevant (*P*=.05). The aggregated average results (SD) between all raters in tasks I and II were also reported as Pearson *χ*^2^. The interrater correlation was determined using Fleiss-Kappa values, following Landis and Koch [22]. The protocol and a first clinical review of the topic have been published before [1,11].

## Results

### Demographics

The mean age in tasks I and II was 51.6 years (SD 13.1), and the experts were significantly younger than the laypersons (mean age 43.6 years, SD 5.7, vs 59.5 years, SD 13.8; *P*=.009). Sex was equally distributed between the groups (total: n=7, 44%, females; n=9, 56%, males; experts: n=4, 50%, females and n=4, 50%, males; laypersons: n=3, 38%, females, and n=5, 62%, males; *P*=.61). All levels of education were present in the laypersons (teachers, housewives, and also academics), whereas the expert group was dominated by professionals in health care, with various qualification levels (residents, consultants), specialties (surgeons, anesthesiologists, internal medicine), and IT specialists (digital transformation officers, software developers). Data are displayed in Table 1.

**Table 1:** Demographic data and summary scores of expert and layperson groups.

Characteristics			Task I									Task II						
			Total (N=16)		Experts (n=8)		Laypersons (n=8)		*P* value		Total (N=16)			Experts (n=8)		Laypersons (n=8)		*P* value
Age (years), mean (SD)			51.6 (13.1)		43.6 (5.7)		59.5 (13.8)		.009		51.6 (13.1)			43.6 (5.7)		59.5 (13.8)		.009
Sex: female, n (%)/male, n (%)			7 (44)/9 (56)		4 (50)/4 (50)		3 (38)/5 (62)		.61		7 (44)/9 (56)			4 (50)/4 (50)		3 (38)/5 (62)		.61
Trustworthiness checklist/APS^a^ survey score, mean sum (SD)			51.0 (2.3)		51.3 (2.7)		50.8 (2.1)		.60		53.1 (0.7)			52.9 (0.8)		53.3 (0.7)		.66
**MARS^b^, mean (SD)**																		
	Engagement summary	4.3 (0.4)		4.5 (0.2)		4.2 (0.4)		.34		4.3 (0.2)			4.3 (0.2)		4.3 (0.2)		.27	
	Functionality summary	4.7 (0.5)		4.8 (0.5)		4.6 (0.5)		.33		4.5 (0.5)			4.6 (0.5)		4.3 (0.5)		.99	
	Aesthetic summary	4.8 (0.4)		4.8 (0.4)		4.7 (0.5)		.69		4.5 (0.2)			4.5 (0.3)		4.5 (0.3)		.99	
	Information summary	4.6 (0.3)		4.5 (0.3)		4.6 (0.2)		.48		4.4 (0.2)			4.4 (0.2)		4.5 (0.1)		.06	
	Subjective quality	4.3 (0.4)		4.5 (0.3)		4.2 (0.5)		.54		4.6 (0.4)			4.6 (0.4)		4.6 (0.4)		.72	
	Perceived impact	4.6 (0.4)		4.9 (0.2)		4.4 (0.3)		.04		4.9 (0.3)			4.9 (0.2)		4.8 (0.3)		.35	
	App quality score^c^	4.6 (0.3)		4.6 (0.2)		4.5 (0.3)		.67		4.4 (0.2)			4.5 (0.2)		4.4 (0.2)		.35	

^a^APS: Aktionsbündnis für Patientensicherheit (alliance for patient safety). The sum of the combined trustworthiness checklist/APS survey scores, with a theoretical maximum score of 62 reflecting answers only with yes. Scoring was performed to achieve comparability between the participants in this study.

^b^MARS: Mobile App Rating Scale. MARS scoring reflects 5 categories of scoring calculated as means (SDs) of the items per category.

^c^The app quality score was calculated as follows: (engagement + functionality + aesthetics + information)/4.

### Raw Data of the Standardized Questionnaires

MARS summaries (engagement: task I mean score 4.3, SD 0.4, and task II mean score 4.3, SD 0.2; functionality: task I mean score 4.7, SD 0.5, and task II mean score 4.5, SD 0.5; aesthetics: task I mean score 4.8, SD 0.4, and task II mean score 4.5, SD 0.2; information: task I mean score 4.6, SD 0.3, and task II mean score 4.4, SD 0.2; subjective quality: task I mean score 4.3, SD 0.4, and task II mean score 4.6, SD 0.4; perceived impact: task I mean score 4.6, SD 0.4, and task II mean score 4.9, SD 0.2; app quality: task I mean score 4.6, SD 0.3, and task II mean score 4.4, SD 0.2) reached high levels (maximum score=5) and were not different between groups, as shown in Table 1 for tasks I and II. The trustworthiness checklist/APS survey mean scores reached values of 51 (SD 2.3) for task I and 53.1 (SD 0.7) for task II for all participants and were not different between experts and laypersons (*P*=.66). Data are displayed in Tables 1 and 2.

**Table 2:** MARS^a^ scoring.

Items and content		Task I rating of scales: experts vs laypersons, *P* value	Task II rating of scales: experts vs laypersons, *P* value
**(A) Entertainment**			
	MARS 1: Is the app fun/entertaining to use? Does it use any strategies to increase engagement through entertainment (eg, through gamification)?	.51	.03
	MARS 2: Is the app interesting to use? Does it use any strategies to increase engagement by presenting its content in an interesting way?	.18	.15
	MARS 3 (customization): Does it provide/retain all necessary settings/preferences for app features (eg, sound, content, notifications)?	.26	.04
	MARS 4 (interactivity): Does it allow user input, provide feedback, contain prompts (reminders, sharing options, notifications, etc)? Note: These functions need to be customizable, not overwhelming to be perfect.	.22	.02
	MARS 5 (target group): Is the app content (visual information, language, design) appropriate for your target audience?	.33	.33
**(B) Functionality**			
	MARS 6 (ease of use): How easy is it to learn how to use the app? How clear are the menu labels/icons and instructions?	.33	.99
**(C) Aesthetics**			
	MARS 7 (layout): Is the arrangement and size of buttons/icons/menus/content on the screen appropriate or zoomable, if needed?	.08	.33
	MARS 8 (graphics): How high is the quality/resolution of graphics used for buttons/icons/menus/content?	.99	.56
	MARS 9 (visual appeal): How good does the app look?	.99	.99
**(D) Information**			
	MARS 10 (accuracy of app description): Does app contain what is described?	.15	.51
	MARS 11 (goals): Does app have specific, measurable, and achievable goals (specified in the app store description or within the app itself)?	<.001	.33
	MARS 12 (quality of information): Is the app content correct, well written, and relevant to the goal/topic of the app?	<.001	<.01
	MARS 13 (quantity of information): Is the extent of coverage within the scope of the app and comprehensive but concise?	.05	.15
	MARS 14 (visual information): Is visual explanation of concepts—through charts, graphs, images, videos, etc—clear, logical, correct?	.23	.04
	MARS 15 (credibility): Does the app come from a legitimate source (specified in the app store description or within the app itself)?	.49	.35
**(E) App subjective quality**			
	MARS 16: Would you recommend this app to people who might benefit from it?	.23	.23
	MARS 17: How many times do you think you would use this app in the next 12 months if it was relevant to you?	.99	.23
	MARS 18: Would you pay for this app?	.23	.99
	MARS 19: What is your overall star rating of the app?	.15	.15
**(F) Perceived impact**			
	MARS 20 (awareness): This app is likely to increase awareness of the importance of addressing prehabilitation.	.01	<.001
	MARS 21 (knowledge): This app is likely to increase knowledge/understanding of prehabilitation.	.99	.99
	MARS 22 (attitudes): This app is likely to change attitudes toward improving prehabilitation.	<.001	.99
	MARS 23 (intention to change): This app is likely to increase intentions/motivation to address prehabilitation.	.03	.99
	MARS 24 (help seeking): Use of this app is likely to encourage further help seeking for prehabilitation (if it is required).	.99	.99
	MARS 25 (behavior change): The use of this app is likely to increase health behaviors with prehabilitation.	<.001	.04

^a^MARS: Mobile App Rating Scale.

### Agreement Within MARS Scoring

MARS scoring was evaluated for internal consistency and compared in a grouped fashion of categories. The internal consistency of the evaluation of raters (Cronbach α) was .77 (range 0.61-0.88) for all raters (N=16), displaying acceptable agreement; .60 (range 0.31-0.79) for the expert group (n=8, 50%), reflecting questionable internal consistency; and .74 (range 0.55-0.86) for the laypersons (n=8, 50%), reflecting acceptable internal consistency in task I [23]. The app quality mean score, calculated as (mean A + mean B + mean C + mean D)/4, was 4.6 (SD 0.2) for the experts and 4.5 (SD 0.3) for the laypersons (*P*=.67). The internal consistency of the evaluation of raters (Cronbach α) was .94 (range 0.91-0.97) for all raters (N=16), displaying excellent agreement, and .89 (range 0.81-0.94) for the experts (n=8, 50%) and .90 (range 0.82-0.95) for the laypersons (n=8, 50%), reflecting good internal consistency between the raters in both groups in task II. Again, the app quality mean score calculated was 4.5 (SD 0.2) for the experts and 4.4 (SD 0.3) for the laypersons (*P*=.35). Notably, the quality was regarded to be high as most values were well above 4 out of 5 possible scoring points (Tables 1 and 2).

### Agreement Within the Trustworthiness Checklist/APS Survey

The trustworthiness checklist/APS survey scoring and questions were analyzed in detail. Data are displayed as Pearson *χ*^2^ evaluating the difference between laypersons’ and experts’ scoring of items (Table 3). Notably, “constants” for tasks I and II reflected a “yes” by all evaluators in both groups and total agreement. The interrater correlation represented by the Fleiss-Kappa values, in accordance with Landis and Koch [22], was 0.72 (range 0.69-0.75) for all raters (N=16), 0.69 (range 0.65-0.74) for the experts (n=8, 50%), and 0.75 (range 0.71-0.79) for the laypersons (n=8, 50%), reflecting substantial agreement between the raters [22]. The mean trustworthiness checklist/APS survey score was 51.3 (SD 2.7) for the experts and 50.8 (SD 2.1) for the laypersons (*P*=.68) in task I. The interrater correlation was 0.87 (range 0.85-0.89) for all raters (N=16), 0.86 (range 0.82-0.91) for the experts (n=8, 50%), and 0.88 (range 0.84-0.93) for the laypersons (n=8, 50%), reflecting almost perfect agreement between the raters. The mean trustworthiness checklist/APS survey score was 52.9 (SD 0.8) for the experts and 53.3 (SD 0.7) for the laypersons (*P*=.66) in task II. Data are displayed in Tables 1 and 3.

**Table 3:** Trustworthiness checklist/APS^a^ survey scoring and questions. Degrees of freedom=1; displayed are *P* values of two sided testing.

Items and content		Task I rating of scales: experts vs laypersons, Pearson *χ*^2^	Task II rating of scales: experts vs laypersons, Pearson *χ*^2^
**Background of the participant**			
	Q ^b^1. Do you often work with medical apps (on a smartphone or personal computer)?	0.25	0.41
	Q2. Have you worked with similar devices (smartphone plus watch)? What were the measurements?	0.52	0.25
**Information accuracy**			
	Q3. Does the app provide accurate measurements?	Constant	Constant
	Q4. Does the app inform end users about errors in measurements?	0.14	Constant
	Q5. Does the app ensure that personalized data tailored to end users are precise?	Constant	Constant
	Q6. Is the information on the app certified by an external source?	Constant	Constant
	Q7. Is the information provided by the app backed by robust research?	Constant	Constant
	Q8. Does the app recommend regular updates?	0.13	Constant
**Understandability**			
	Q9. Is the app accompanied by clear end-user safety guidelines?	Constant	Constant
	Q10. Is research-backed evidence used to create the app easy to locate and understand?	Constant	Constant
**Transparency**			
	Q11. Does the app highlight potential risks or side effects resulting from its use?	Constant	Constant
	Q12. Are the terms of service concise and easy to read?	0.13	Constant
	Q13. Does the app require only minimal personal data of end users?	Constant	Constant
	Q14. Are the privacy policies concise, clear, and easy to understand?	Constant	Constant
**Brand familiarity**			
	Q15. Does the company have other reputable products or services to associate the app with?	Constant	Constant
**Reputation**			
	Q16. Does the company curating the app have clear policies on how to handle end-user data?	Constant	Constant
	Q17. Does the company make its data-handling history and data breaches available to end users?	Constant	Constant
	Q18. Is the app affiliated with a nongovernmental organization or a reputable government agency?	Constant	0.52
	Q19. Does the company value data protection regulations?	0.06	Constant
	Q20. Does the company use skilled personnel within the app development domain?	Constant	Constant
**External factor**			
	Q21. Does the app accompany a wearable device?	Constant	Constant
**Usability**			
	Q22. Is the app easy to use, and does it have a friendly end-user interface?	Constant	Constant
	Q23. Is the app visually appealing (aesthetics)?	Constant	Constant
	Q24. Does the app send out a reasonable number of notifications?	Constant	Constant
	Q25. Are the features of the app customizable?	0.05	Constant
	Q26. Is the app accessible by its target audience?	1.00	0.06
**Privacy**			
	Q27. Is privacy a core consideration throughout the app design phase (ie, a privacy-by-design approach)?	Constant	Constant
	Q28. Are the data generated from the app pseudonymized so that individuals are not easily identifiable?	Constant	Constant
	Q29. Can users easily access all their data (eg, address, billing information)?	1.00	Constant
	Q30. Do the functions of the app give end users the overall impression of freedom to control the use of their data?	Constant	Constant
**Empowerment**			
	Q31. Does the app allow end users to restrict data sharing to third parties, such as social networking sites?	Constant	Constant
	Q32. Does the app allow end users to opt in and decide which data can be stored or processed?	0.13	Constant
	Q33. Does the app allow end users to easily delete their data?	1.00	Constant
**Purpose and functionality**			
	Q34. The specific scope of the app is clearly described.	Constant	Constant
	Q35. The app lists its own limitations (eg, a disclaimer stating that the app cannot replace a real-life medical consultation).	Constant	Constant
	Q36. The app only requests data that are important to its functionality.	Constant	Constant
	Q37. Requests for access to functions of the mobile device (eg, access to location via the GPS or to the calendar) are only requested to facilitate app usage.	Constant	Constant
**Quality and evaluation**			
	Q38. The app does not provide a definitive diagnosis and corresponding treatment recommendations.	1.00	0.30
	Q39. Does the app ask for baseline data?	Constant	Constant
	Q40. The app does support existing treatment plans (eg, by recording biometrics).	Constant	Constant
**Technical aspects**			
	Q41. The app allows sharing of data.	0.13	Constant
	Q42. The app has an app community.	0.30	Constant
	Q43. The app allows password protection.	Constant	Constant
	Q44. The app requires a login.	Constant	Constant
	Q45. The app sends reminders to the patient and the doctor.	Constant	Constant
	Q46. The app needs web access to function.	Constant	Constant
**Embedded in therapeutic concepts**			
	Q47. Not embedded in a clear therapeutic concept.	0.30	Constant
	Q48. The patient can communicate with the doctor via the app.	Constant	Constant
	Q49. The doctor can communicate with the patient via the app.	Constant	Constant
	Q50. Results are shared between doctor and patient.	Constant	Constant
	Q51. The app has terms of use.	Constant	Constant
	Q52. The app has a privacy policy statement (APS).	Constant	Constant
	Q53. The app has a legal notice and liability statement.	Constant	Constant
	Q54. The app has data safety and data storage information and agreements (APS).	Constant	Constant
	Q55. The app requires active informed consent.	Constant	Constant
	Q56. The app has an emergency function/contact.	Constant	Constant
	Q57. The app provides information on data sharing with third parties (APS).	0.59	Constant
	Q58. The information is easy to find and view (APS).	Constant	Constant
	Q59. The app provides information on how to withdraw consent for data storage (APS).	Constant	Constant
	Q60. The app specifies that it is possible for collected and stored data to be deleted (APS).	0.30	Constant
**Imprint contains the following**			
	Q61. The app contains the name and address of the provider. Legal entities (eg, Inc, PLC, Ltd, Co), in particular, must state their legal form and authorized representatives.	Constant	Constant
	Q62. The app contains details for direct and immediate contact (telephone or fax number, email address).	Constant	Constant
**Funding and financial background**			
	Q63. The costs for the app are clearly explained in the user manual.	1.00	0.52
	Q64. The app is provided by a public or charitable organization.	0.30	0.36

^a^APS: Aktionsbündnis für Patientensicherheit (alliance for patient safety).

^b^Q: question.

### Usability Testing and Alarm Recognition

The experts (doctors) and laypersons (patients and potential patients) had to face a total of 11 scenarios, 5 (45%) doctor-specific scenarios and 6 (55%) patient-specific scenarios. A set of 3 answers was provided related to satisfaction with the ease of completion, timely satisfaction, and support satisfaction (scale from 1=strongly agree to 7=strongly disagree); each evaluator chose “done with ease,” “done with problems,” or “failed.” The duration for the different scenarios is displayed in Table 4 for tasks I and II. As hypothesized, most doctors did not have any problems with usability scenarios D1, D2, D4, and D5, reflected by low mean values for the satisfaction of ease, timely satisfaction, and support satisfaction with the scenarios, and the evaluator found good performance (done with ease) for all 8 doctors. Only usability scenario D3, which required launching the app on the patient’s smartphone, onboarding the patient, and presenting the workflow to a virtual patient was associated with problems and required an average of >20 minutes per doctor. Similar results were found in the laypersons’ usability scenarios, where usability scenario P1 required onboarding by the patient. Herem the findings were the same: with some problems and a similar duration of >20 minutes. Similarly, laypersons were satisfied with usability scenarios P2-P6 in most cases and had high satisfaction rates. Data of tasks I and II are displayed in Table 4.

**Table 4:** Usability scenarios for experts (doctors) and laypersons (patients and potential patients).

Scenario	Usability scenario	Duration (seconds), mean (SD)	Satisfaction with ease of completion, mean (SD)	Timely satisfaction, mean (SD)	Support satisfaction, mean (SD)	Evaluator, mean (SD)
D^a^1	Create a new patient and enter risk assessment data.	211 (13)	1.50 (0.53)	1.50 (0.53)	1.38 (0.52)	1.00 (0)
D2	Correct patient data if an error has occurred.	103 (38)	1.25 (0.46)	1.63 (0.52)	1.00 (0)	1.00 (0)
D3	Launch the system on the patient’s smartphone, the onboarding, and the workflow presentation.	1213 ±191)	2.75 (0.46)	3.38 (0.52)	3.5 (0.53)	2.0 (0)
D4	Notification and reaction to safety audit.	77 (6)	1.25 (0.46)	1.25 (0.46)	1.25 (0.46)	1.00 (0)
D5	Detect the patient in the follow-up list and perform 90-day follow-up.	147 (11)	1.50 (0.53)	1.38 (0.52)	1.38 (0.52)	1.00 (0)
P^b^1	Safe onboarding and smartwatch connection.	1266 (136)	3.13 (0.35)	3.38 (0.52)	3.38 (0.74)	2.0 (0)
P2	Dashboard and content exploration.	220 (26)	1.88 (0.35)	1.63 (0.52)	1.63 (0.52)	1.00 (0)
P3	Launch the 6MWT^c^ or exercise.	78 (16)	1.50 (0.53)	1.63 (0.52)	1.63 (0.52)	1.00 (0)
P4	Fill out postexercise patient-reported outcome measures (PROMs).	47 (15)	1.38 (0.52)	1.38 (0.52)	1.13 (0.35)	1.00 (0)
P5	Notification and reaction to safety audit.	116 (20)	1.88 (0.35)	1.75 (0.46)	1.25 (0.46)	1.00 (0)
P6^d^	Reaction to haptic and optical alarm on smartwatch.	10 (8)	2.03 (1.32)	1.97 (1.31)	1.99 (1.31)	1.41 (0.57)

^a^D: doctor.

^b^P: patient.

^c^6MWT: 6-Minute Walk Test.

^d^Usability scenario P6 was carried out on the treadmill for 75 patients in task IIIb of the clinical investigation.

### Personal Comments of Experts and Laypersons

All experts and laypersons were asked to select their top 3 best items and top 3 items requiring improvement after the task I mockup evaluation. The most frequent terms related to items deemed the best were “good instructions,” “intuitive,” “dashboard” and “dashboard functions,” “simple,” “clear,” “design and layout,” “structured risk assessment,” “outcome data,” “benchmarking,” “thresholds,” “understandable,” “aims and structure,” “design,” and “fat finger buttons.” The most frequent terms used for things that could still be improved were “the length of the user manual,” “more items for individualization,” “more information on other aspects of prehabilitation,” “nutrition,” “specify heart rate details,” “a quick start option,” “infographics,” “different workflows,” “for other indications,” and “hospital journey.”

## Discussion

### Principal Findings

The formative and summative evaluation of the Prehab App design resulted in good-to-acceptable results of the design and usability of the critical and safety-relevant areas of the medical device and stand-alone software. With this approach, we follow a strict usability engineering process required by the MDR and as defined in DIN ISO 62366:2017. We were able to design a “safety by design” interface that avoids errors and hazardous situations and tested those scenarios in this clinical investigation. To date, the workflow of the pathway seems to be logical and without any dead ends and complicated usability issues. There was high quality reflected by high values constantly above 4 in MARS and values of 50 and higher in the trustworthiness checklist/APS survey. Interrater and intrarater reliabilities of the different tests were acceptable, substantial, and almost perfect. The usability scenarios detected the onboarding and connection process of the software to the wearable as a specific area that requires further attention to set up a guided and smooth workflow, which is critical for the penetration and success of software that should be used in a remote setting.

Following the regulatory pathway was of critical importance for the research group from the beginning. As Capreolos GmbH is a spin-off of Goethe University due to the necessity to build up a DIN ISO 13485– and MDR-compliant quality management, the avoidance of a quality chasm was a major milestone from the start. We managed this by seeking counseling from different experts and advisors: (1) first, an experienced business angel who is a founder and regulatory expert in the field for more than 25 years advised us from the beginning; (2) second, a chair of a German university–based independent ethical review board accompanied the early phase of building the company with their regulatory advice, further connecting us to an external data protection specialist; and (3) third, a professional software regulatory consulting company allocated a dedicated single contact person to the group, which was of high value from the beginning, starting in 2021. Having a team of developers and cofounders, as well as a research group that understood and embraced the regulatory requirements of DIN ISO 13485, DIN ISO 14155, the MDR, and the MPDG, with all their accompanying requirements, we were able to set up the current program in a translational, academic, regulatory-compliant approach.

One of the major challenges in the design of a smart device is not only to consider manual usability that suits the technical skills of the users but also to design interfaces and workflows that provide smart and simple pathways through the app, avoid mistakes, and not lead to frustration on the users’ side, as outlined by other groups and indications before [24,25]. By targeting the right population and with a clear definition of the intended use, respecting the special needs of the target population, and fostering autonomy, as well as communication strategies, a high level of adherence and compliance becomes likely and can be implemented in a perioperative interventional app–based solution [26,27]. This is especially important as the Prehab App will be used in a remote setting, fostering patient empowerment and autonomy using the short-term motivation after a cancer diagnosis to be as fit as possible for the operation. A survey among patients and experts showed that the willingness to use a digital intervention is high before major surgeries if usability is smart and simple by otherwise avoiding excessive notifications and push messages generating alarm fatigue and demotivation [9,28-30]. The success of a digital solution, thus, depends not only on a great design but also on how to engage users on socioeconomic and motivational levels [31]. To give an example, for the specific app in this study, success could mean a high conversion rate of patients from vulnerable to less vulnerable before surgery. On the doctors’ side, it could be the ratio of compliantly exercising patients delivering better outcomes. On the patients’ side, an easy-to-accomplish program that generates fun and mental well-being before major surgeries, including tips for optimal nutrition, may increase motivation and lead to improvements in physical health and reduce morbidity after surgery.

MARS and the trustworthiness checklist modified by the APS online checklist are suitable tools to assess the usability of mockups and have repeatedly proven to have a good correlation in the literature for both experts and laypersons [15,20,32]. Although the tools were initially designed for expert use, there are convincing data that they work similarly in the hands of patients.

### Strengths

A considerable strength of the current usability assessment is that a clear regulatory requirement was followed and that the study groups had a good mix of experienced academic researchers, young digital health care enthusiasts, economically thinking surgeons, and developers with the right view for design, look and feel, and usability. All scenarios were based on DIN ISO 14971–compliant risk management and covered the critical and most dangerous aspects for patient safety. However, the most critical finding and challenge during the usability scenarios was the smooth onboarding pathway. Both experts and laypersons struggled in this scenario. Although this is completely unrelated to the software, the connection of a wearable to the software must be ensured by the manufacturer of the software if it has to be used flawlessly. The observation is a common problem with digital wearables, as this may be a potential showstopper and has been repeatedly reported to be associated with reduced compliance and satisfaction of patients and other users [33,34]. To mitigate the risk of noncompliance and frustration, special onboarding teams may serve as help for the teams and provide ongoing expert company, especially in the early phase of going live at a new site. In the latter setting, it is not necessary to completely onboard the patient in the hospital, as they can do this at home remotely. Notably, we explicitly decided to use commercially available wearables, and the setup process is a well-described publicly available process that has been successfully achieved by hundreds of millions of people in all age groups worldwide, which should be a promising basis for later success with the right support.

### Limitations

The general weakness of the usability trial may be that there was no repetition of the scenarios by the same person. However, there were few failures in single scenarios, and it was not within the scope of this study to test the effects of getting used to repetitive walkthroughs of the same scenarios. Usability was tested in a simulated setting mimicking the environment as much as possible. However, the scenarios were not tested in a remote setting, because safety testing was more important than testing the remote application. The risk of serious usability safety issues could be mitigated by this trial.

### Future Implications

The first data from the clinical application have further added to the body of evidence [10,12]. After adjusting the risk management following DIN ISO 14971 and updating the clinical evaluation following the MDR and DIN ISO 13845, we are already in the final preparations of an RCT to test the safety and efficacy of the Prehab App in a remote setting with approximately 400 patients.

The criticisms raised by the users, such as more information about certain aspects of prehabilitation, individualization, the length of the user manual, and infographics, have already been considered in the app’s version 2 and were successfully implemented.

### Conclusion

This usability trial of the Prehab App was successful and revealed good usability of the app’s design. In combination with the clinical data obtained, the most critical risks identified in the risk management could be mitigated. The initiation of an RCT on prehabilitation in a remote setting with measured interventions at home seems feasible and ethically and medically justified.

## Data Availability

We do not routinely provide complete data sets. However, if a secondary research question arises, we will decide internally to provide an anonymized data set for analysis.

## Appendix

Multimedia Appendix 1 Task II survey for experts and patients: use scenarios.

## Abbreviations

6MWT: 6-Minute Walk Test
APS: Aktionsbündnis für Patientensicherheit (alliance for patient safety)
ASQ: after scenario questionnaire
DIN: Deutsches Institut fur Normung (German industry norm)
ECG: electrocardiogram
ISO: International Organization for Standardization
MARS: Mobile App Rating Scale
MDR: Medical Device Regulation
MPDG: Medizinproduktedurchführungsgesetz (medical devices implementation law)
MVP: minimal viable product
RCT: randomized controlled trial
