# Supplementary material for: Usability Testing of a Digitized Interventional Prehabilitation Tool for Health Care Professionals and Patients Before Major Surgeries: Formative and Summative Evaluation
Source: JMIR Form Res. 2024 Dec 11;8:e59513. doi: 10.2196/59513 (PMC11669884; doi:10.2196/59513)
Supplement: Multimedia Appendix 1 [file formative_v8i1e59513_app1.docx]

**Multimedia Appendix 1. Task II survey for experts and patients: use scenarios.**

**Evaluation of the Patronus Prehab App for usability testing using heuristic scenarios and specific tasks reflecting the intended use and an intuitive and safe workflow for doctors and patients.**

Instruction to Evaluator:

1. Please review the app, and the app description inside the app covering the intended use, the target group as well as data safety and privacy, and the user manual. Thereafter, please answer the following questions on app usability, functionality and trustworthiness using the structured questionnaires.
2. Please use the App and follow the use scenarios. An authorized person will explain the task to you, observe you by accomplishing the task and document the time you need for the use scenarios to accomplish.
3. Please fill out the ASQ after every single use scenario.

App-Name: The Prehab App

Task II: Rating the Life Version (finalized iterative process of the design development based on the specification book) and Heuristic Use Scenarios for Doctors and Patients

Developer: Capreolos GmbH, Frankfurt

Individuals rating this version: 8 professionals and experts, 8 potential patients

Version: 2, Oct. 14^th^, 2021

Please answer the following questions before you start:

Date of evaluation: DD.MMM.YYYY

Evaluator ID: pseudonymized

Supervisor ID: name

Job title: give profession

Do you work with such devices often? Y/N

Have you worked with similar devices= Y/N

Name the top 3 things you would change in such devices:

What are the top 3 things you like most about such devices?

**I: Trustworthiness and APS checklist**

| **General characteristics** | | **Select all that apply** | **Yes** | **No** |
| --- | --- | --- | --- | --- |
| Information Accuracy | | Does the app provide accurate measurements? |  |  |
|  |  | Does the app inform end-users about errors in measurements? |  |  |
|  |  | Does the app ensure that personalised data tailored to end-users are precise? |  |  |
|  |  | Is the information on the app certified by an:  a. in-house team? |  |  |
|  |  | b. external third-party team? |  |  |
|  |  | Is the information provided by the app backed by robust research? |  |  |
|  |  | Does the app recommend regular updates to:  a. fix bugs inherent within the app? |  |  |
|  |  | b. amend app contents based on improved  research? |  |  |
| Understandability | | Is the app accompanied by clear end-user safety guidelines? |  |  |
|  |  | Is the research-backed evidence used to create the app easy to locate and understand? |  |  |
| Transparency | | Does the app highlight potential risks or side effects resulting from its use? |  |  |
|  |  | Are the ‘terms of service’ concise and easy to read? |  |  |
|  |  | Does the app require only minimal personal data of end-users?* |  |  |
|  |  | Are the privacy policies concise, clear and easy to understand? |  |  |
| Brand familiarity | | Does the company have other reputable products or services to associate the app with? |  |  |
| Reputation | | Does the company curating the app have clear policies on how to handle end-user data? |  |  |
|  |  | Does the company make their data handling history and data breaches available to end-users? |  |  |
|  |  | Is the app affiliated with a non-governmental organization or a reputable government agency?* |  |  |
|  |  | Does the company value data protection regulations? |  |  |
|  |  | Does the company utilise skilled personnel within the app development domain? |  |  |
| External factor |  | Does the app accompany a wearable device? |  |  |
| Usability | | Is the app easy to use and have a friendly end-user interface? |  |  |
|  |  | Is the app visually appealing (aesthetics)? |  |  |
|  |  | Does the app send out a reasonable number of notifications?* |  |  |
|  |  | Are the features of the app customisable? |  |  |
|  |  | Is the app accessible by its target audience?* |  |  |
| Privacy | | Is privacy a core consideration throughout the app  design phase, i.e. a privacy by design approach? |  |  |
|  |  | Is the data generated from the app pseudonomised so that individuals are not easily-identifiable? |  |  |
|  |  | Can users easily access all of their data e.g. address, billing information? |  |  |
|  |  | Is privacy a core consideration throughout the app design phase, i.e. a privacy by design approach? |  |  |
|  |  | Do the functions of the app give end-users the overall impression of freedom to control the use of their data? |  |  |
| Empowerment | | Does the app allow end-users to restrict data sharing to third-parties such as social networking sites? |  |  |
|  | | Does the app allow end-users to opt-in and decide which data can be stored or processed?* |  |  |
|  | | Does the app allow end-users to easily delete their data? |  |  |
| Purpose and Functionality (APS) | | The specific scope of the app is clearly described |  |  |
|  |  | The app lists its own limitations (e.g. disclaimer stating that the app cannot replace a real-life medical consultation). |  |  |
|  |  | The app only requests data that are important to its functionality. |  |  |
|  |  | Requests for access to functions of the mobile device (e.g. access to location via GPS or to the calendar) are only requested in order to facilitate app usage. |  |  |
| Quality and Evaluation  (APS) | | The app does not provide a definitive diagnosis and corresponding treatment recommendations. |  |  |
|  |  | Does the app ask for baseline data? |  |  |
|  |  | The app does support existing treatment plans (e.g. by recording biometrics). |  |  |

| **General characteristics** | **Select all that apply** | **Yes** | **No** |
| --- | --- | --- | --- |
| Technical Aspects of App | Allows sharing of data |  |  |
|  | Has an app community |  |  |
|  | Allows password protection |  |  |
|  | Requires a login |  |  |
|  | Sends reminders to the patient and the doctor |  |  |
|  | Needs web access to function |  |  |
| Embedded in therapeutic concepts | Not embedded in a clear therapeutic concept |  |  |
|  | Patient can communicate with doctor via app |  |  |
|  | Doctor can communicate with patient via app |  |  |
|  | Results are shared between doctor and patient |  |  |
|  | The app has “Terms of Use” |  |  |
|  | The app has a privacy policy statement (APS) |  |  |
|  | The app has a legal notice and liability statement |  |  |
|  | The app has a data safety and data storage information and agreement (APS) |  |  |
|  | The app requires active informed consent |  |  |
|  | The app has an emergency function/contact |  |  |
|  | The app gives information on data sharing with third parties (APS) |  |  |
|  | The information is easy to find and to view (APS) |  |  |
|  | The app gives information on how to withdraw consent for data storage (APS) |  |  |
|  | The app specifies that it is possible for collected and stored data to be deleted. (APS) |  |  |
| Imprint contains the following (APS) | The name and address of the provider. Legal entities (e.g. Inc., PLC, Ltd., Co.) in particular must state their legal form and authorized representatives. |  |  |
|  | Details for direct and immediate contact (telephone or fax number, email address). |  |  |
| Funding and financial background (APS) | The costs for the app are clearly explained in the user manual. |  |  |
|  | The app is provided by a public or charitable organization |  |  |

**II. MARS (Mobile Application Rating Scale)**

| **Is the app fun/entertaining to use? Does it use any strategies to increase engagement through entertainment (e.g. through gamification)?** | - 1 Dull, not fun or entertaining at all - 2 Mostly boring - 3 OK, fun enough to entertain user for a brief time (< 5 minutes) - 4 Moderately fun and entertaining, would entertain user for some time (5-10 min) - 5 Highly entertaining and fun, would stimulate repeat use |
| --- | --- |
| **Is the app interesting to use? Does it use any strategies to increase engagement by presenting its content in an interesting way?** | - 1 Not interesting at all - 2 Mostly uninteresting - 3 OK, neither interesting nor uninteresting; would engage user for a brief time (< 5 min) - 4 Moderately interesting; would engage user for some time (5-10 minutes total) - 5 Very interesting, would engage user in repeat use |
| **Customisation: Does it provide/retain all necessary settings/preferences for apps features (e.g. sound, content, notifications, etc.)?** | - 1 Does not allow any customisation or requires setting to be input every time - 2 Allows insufficient customisation limiting functions - 3 Allows basic customisation to function adequately - 4 Allows numerous options for customisation - 5 Allows complete tailoring to the individual’s characteristics/preferences |
| **Interactivity: Does it allow user input, provide feedback, contain prompts (reminders, sharing options, notifications, etc.)? Note: these functions need to be customisable and not overwhelming in order to be perfect.** | - 1 No interactive features and/or no response to user interaction - 2 Insufficient interactivity, or feedback, or user input options, limiting functions - 3 Basic interactive features to function adequately - 4 Offers a variety of interactive features/feedback/user input options - 5 Very high level of responsiveness through interactive features/feedback/user input options |
| **Target group: Is the app content (visual information, language, design) appropriate for your target audience?** | - 1 Completely inappropriate/unclear/confusing - 2 Mostly inappropriate/unclear/confusing - 3 Acceptable but not targeted. May be inappropriate/unclear/confusing - 4 Well-targeted, with negligible issues - 5 Perfectly targeted, no issues found |
| **Ease of use: How easy is it to learn how to use the app; how clear are the menu labels/icons and instructions?** | - 1 No/limited instructions; menu labels/icons are confusing; complicated - 2 Useable after a lot of time/effort - 3 Useable after some time/effort - 4 Easy to learn how to use the app (or has clear instructions) - 5 Able to use app immediately; intuitive; simple |
| **Layout: Is arrangement and size of buttons/icons/menus/content on the screen appropriate or zoomable if needed?** | - 1 Very bad design, cluttered, some options impossible to select/locate/see/read - 2 Bad design, random, unclear, some options difficult to select/locate/see/read - 3 Satisfactory, few problems with selecting/locating/seeing/reading items size problems - 4 Mostly clear, able to select/locate/see/read items - 5 Professional, simple, clear, orderly, logically organised, device display optimised. Every design component has a purpose |

| **Graphics: How high is the quality/resolution of graphics used for buttons/icons/menus/content?** | - 1 Graphics appear amateur, very poor visual design - disproportionate, completely stylistically inconsistent - 2 Low quality/low resolution graphics; low quality visual design – disproportionate, stylistically inconsistent - 3 Moderate quality graphics and visual design (generally consistent in style) - 4 High quality/resolution graphics and visual design – mostly proportionate, stylistically consistent - 5 Very high quality/resolution graphics and visual design - proportionate, stylistically consistent throughout |
| --- | --- |
| **Visual appeal: How good does the app look?** | - 1 No visual appeal, unpleasant to look at, poorly designed, mismatched colours - 2 Little visual appeal – poorly designed, bad use of colour, visually boring - 3 Some visual appeal – average, neither pleasant, nor unpleasant - 4 High level of visual appeal/seamless graphics/consistent and professionally designed - 5 As above+very attractive, memorable, stands out; use of colour enhances app features/menus |
| **Accuracy of app description: Does app contain what is described?** | - 1 Misleading. App does not contain the described components/functions. Or has no description - 2 Inaccurate. App contains very few of the described components/functions - 3 OK. App contains some of the described components/functions - 4 Accurate. App contains most of the described components/functions - 5 Highly accurate description of the app components/functions |
| **Goals: Does app have specific, measurable and achievable goals (specified in app store description or within the app itself)?** | - N/A Description does not list goals, or app goals are irrelevant to research goal (e.g. using a game for educational purposes) - 1 App has no chance of achieving its stated goals - 2 Description lists some goals, but app has very little chance of achieving them - 3 OK. App has clear goals, which may be achievable. - 4 App has clearly specified goals, which are measurable and achievable - 5 App has specific and measurable goals, which are highly likely to be achieved |
| **Quality of information: Is app content correct, well written, and relevant to the goal/topic of the app?** | - N/A Thereisnoinformationwithintheapp - 1 Irrelevant**/**inappropriate/incoherent/incorrect - 2 Poor. Barely relevant/appropriate/coherent**/**may be incorrect - 3 Moderately relevant/appropriate/coherent**/**and appears correct - 4 Relevant**/**appropriate/coherent/correct - 5 Highly relevant, appropriate, coherent, and correct |
| **Quantity of information: Is the extent coverage within the scope of the app; and comprehensive but concise?** | - N/A There is no information within the app - 1 Minimal or overwhelming - 2 Insufficient or possibly overwhelming - 3 OK but not comprehensive or concise - 4 Offers a broad range of information, has some gaps or unnecessary detail; or has no links to more information and resources - 5 Comprehensive and concise; contains links to more information and resources |

| **Visual information: Is visual explanation of concepts – through charts/graphs/images/videos, etc. – clear, logical, correct?** | - N/A There is no visual information within the app (e.g.it only contains audio or text) - 1 Completely unclear/confusing/wrong or necessary but missing - 2 Mostly unclear/confusing/wrong - 3 OK but often unclear/confusing/wrong - 4 Mostly clear/logical/correct with negligible issues - 5 Perfectly clear/logical/correct |
| --- | --- |
| **Credibility: Does the app come from a legitimate source (specified in app store description or within the app itself)?** | - 1 Source identified but legitimacy/trustworthiness of source is questionable (e.g. commercial business with vested interest) - 2 Appears to come from a legitimate source, but it cannot be verified (e.g. has no webpage) - 3 Developed by small NGO/institution (hospital/centre, etc.) /specialised commercial business, funding body - 4 Developed by government, university or as above but larger in scale - 5 Developed using nationally competitive government or research funding |
| **Would you recommend this app to people who might benefit from it?** | - 1 **Not at all** - 2 I would not recommend this app to anyone - 3 **Maybe** - 4 There are very few people I would recommend this app to There are several people whom I would recommend it to There are many people I would recommend this app to - 5 **Definitely** I would recommend this app to everyone |
| **How many times do you think you would use this app in the next 12 months if it was relevant to you?** | - 1 **None** - 2 1-2 - 3 3-10 - 4 10-50 - 5 >50 |
| **Would you pay for this app?** | - 1 No - 3 Maybe - 5 Yes |
| **What is your overall star rating of the app?** | - 1 ★ - 2 ★★ - 3 ★★★ - 4 ★★★★ - 5 ★★★★★ |
| **Awareness: This app is likely to increase awareness of the importance of addressing [insert target health behaviour]** | - 1 Strongly disagree - 2 Disagree to some extent - 3 Indifferent - 4 Agree to some extent - 5 Strongly agree |
| **Knowledge: This app is likely to increase knowledge/understanding of [insert target health behaviour]** | - 1 Strongly disagree - 2 Disagree to some extent - 3 Indifferent - 4 Agree to some extent - 5 Strongly agree |

| **Attitudes: This app is likely to change attitudes toward improving [insert target health behaviour]** | - 1 Strongly disagree - 2 Disagree to some extent - 3 Indifferent - 4 Agree to some extent - 5 Strongly agree |
| --- | --- |
| **Intention to change: This app is likely to increase intentions/motivation to address [insert target health behaviour]** | - 1 Strongly disagree - 2 Disagree to some extent - 3 Indifferent - 4 Agree to some extent - 5 Strongly agree |
| **Help seeking: Use of this app is likely to encourage further help seeking for [insert target health behaviour] (if it’s required)** | - 1 Strongly disagree - 2 Disagree to some extent - 3 Indifferent - 4 Agree to some extent - 5 Strongly agree |
| **Behaviour change: Use of this app is likely increase/decrease [insert target health behaviour]** | - 1 Strongly disagree - 2 Disagree to some extent - 3 Indifferent - 4 Agree to some extent - 5 Strongly agree |

**Use Scenarios and ASQ**

**Doctor/Qualified Nurse**

**Scenario 1: you are a new user of the Patronus Prehab App. Your hospital admin for the Patronus Prehab App has provided you with an email containing your Username and initial password as well as a link to an Apple/Google-store to download the App and a link to the Desktop version of the App for patient screening.**

**Please, execute the following use scenario task and tell your supervisor when you are ready to take the time.**

1. Download the App to your smartphone
2. Perform onboarding
3. Create a new patient and perform and finish the risk assessment

**ASQ**

**Supervisor evaluation sheet with the expected walkthrough**:

### [UR1] Scenario 1: Onboarding and create new patient

| Core task | <UR1> Onboarding, new patient | Done with ease | Done with problems | Failed |
| --- | --- | --- | --- | --- |
| Short description | Create a new patient and enter the risk assessment data |  |  |  |
| Pre-condition(s) | The patient list does not include the new patient |  |  |  |
| Post-condition(s) | The patient list includes the new patient |  |  |  |
| Regular flow | - Doctor accesses his account - Gets to active patients list - Presses plus sign - Confirms exclusion of contraindications against 6MWT and aerobic moderate exercising - Not ticking the contraindications prohibits to proceed - Answers all questions, queries and warnings - Gets results - Presses finish and gets QR code for patient - Sends QR to his personal email - Presses Finish - Gets back to active patient list |  |  |  |
| Variants alternative flows, exceptions* | Only one workflow allowed. This is a clear A to B to C to D process that does not allow alternatives way. It is possible to go back with < to correct data. | Please document if user found alternative way. | | |

Time required: _____minutes _____seconds

**Scenario 3:**

**You are the treating doctor. In this task you will solve problems with wrong data entry during the risk assessment and after creating the QR code and an exercising program.**

**Please tell your supervisor when you are ready, they will start taking the time and put in the following data for your virtual patient until the end or follow the advice on the app.**

- 1. Go to the basic data pre-program page. Tab on the pencil icon and go to the page where you made a mistake. Correct all data for the patient that you received by the moderator.
  2. Finish the risk assessment

**ASQ**

**Supervisor evaluation sheet with the expected walkthrough**:

### [UR2=UR7] Scenario 3: Wrong data entry correction

| Core task | <UR2> Wrong data entry correction | Done with ease | Done with problems | Failed |
| --- | --- | --- | --- | --- |
| Short description | Correct patient data after a QR code has been generated |  |  |  |
| Pre-condition(s) | Wrong data in a patient after creation of a QR code |  |  |  |
| Post-condition(s) | Corrected patient data |  |  |  |
| Regular flow | - Correct Hgb to 15 - Correct ECOG to 0 - Correct weight to 65kg - Switch to patient is on chemotherapy - Change RAI C items-elaborate a bit on that. |  |  |  |
| Variants alternative flows, exceptions* | Only one workflow allowed. This is a clear A to B to C to D process that does not allow alternatives way. It is possible to go back with < to correct data. It is not possible to proceed, if data are not entered completely. | Please document if user found alternative way. | | |

Time required: _____minutes _____seconds

**Scenario 4:**

**You are the treating doctor. In this task you will perform onboarding with the patient and explain the content of the app and the program to the patient.**

**Please tell your supervisor when you are ready, they will start taking the time and put in the following data for your virtual patient until the end or follow the advices on the app.**

1. Perform onboarding with the patient
2. Explain the functions to the patient
3. Explain the icons to the patient

**ASQ**

**Supervisor evaluation sheet with the expected walkthrough**:

### [UR3=UR8] Scenario 4: Train patient onboarding and app workflow

| Core task | <UR3> Train patient | Done with ease | Done with problems | Failed |
| --- | --- | --- | --- | --- |
| Short description | The doctors launch the system on the patient smartphone, perform onboarding together with the patient, setup smartwatch and show the workflow to the patient. |  |  |  |
| Pre-condition(s) | The patient has a QR code provided by the doctor |  |  |  |
| Post-condition(s) | The patient has an installed app and a ready to use smartwatch and knows the functions of the app they need to use. |  |  |  |
| Regular flow | - QR code is scanned via camera - App is installed - Onboarding and Health data activation - Homescreenoverview with tab bar - Doctor explains dashboard on homescreen - Doctor explains tab bar icons - Doctor gets to exercise overview and explain 6MWT, exercise, QoL and nutrition toggle bars in combination with calendar - Doctor explains that calendar allows review of individual exercise - Doctor explains communication area (alarms, warnings, reminders) - Doctor explains smartwatch screens with training threshold areas and functions - Doctor explains Symptom checklist to patient - Doctor explains Wiki functions - Doctor does not launch an exercise but explains exercise to patient with the user manual |  |  |  |
| Variants alternative flows, exceptions* | Various workflows can be started from the homescreen of the patient. With the home icon a return to the home screen is possible. Tab bars must not be launched in a fixed sequence. | Please document if user found alternative way. | | |

Time required: _____minutes _____seconds

**Scenario 5:**

**You are the treating doctor. In this task you will work on the automated safety audit. Starting point is your home screen.**

**Please tell your supervisor when you are ready, they will start taking the time and put in the following data for your virtual patient until the end or follow the advices on the app.**

1. Access the safety audit
2. Clear all outstanding notifications

**ASQ**

**Supervisor evaluation sheet with the expected walkthrough**:

### [UR4] Scenario 5: Safety audit

| Core task | <UR4> Safety audit | Done with ease | Done with problems | Failed |
| --- | --- | --- | --- | --- |
| Short description | The doctors are notified on the software when there are push-warnings that are potentially safety relevant. |  |  |  |
| Pre-condition(s) | The doctor has unresponded push-notifications and warnings |  |  |  |
| Post-condition(s) | The doctor confirms push-notifications and warnings in a risk-stratified way |  |  |  |
| Regular flow | - The doctor receives messages in the safety audit of the app for every individual patient - The doctor confirms messages |  |  |  |
| Variants alternative flows, exceptions* | The sequence of the patients to review can be chosen by the doctors. Red alerts require immediate action, yellow alerts launch automated responses and notifications to the patient. | Please document if user found alternative way. | | |

Time required: _____minutes _____seconds

**Scenario 7:**

**You are the treating doctor. In this task you will perform the 90-day follow up. Starting point is the home screen.**

**Please tell your supervisor when you are ready, they will start taking the time and put in the following data for your virtual patient until the end or follow the advices on the app.**

1. Access the defined patient.
2. Perform the 90 day follow up.
3. Finish the program for the patient

**ASQ**

**Supervisor evaluation sheet with the expected walkthrough**:

### [UR6=UR10] Scenario 7: Perform 90 day follow up

| Core task | <UR6> 90 day follow up | Done with ease | Done with problems | Failed |
| --- | --- | --- | --- | --- |
| Short description | The doctors are notified when the patient reaches their 90 day follow up date. They contact the patient and fill out the 90 day follow up data. |  |  |  |
| Pre-condition(s) | The final follow up data are not entered |  |  |  |
| Post-condition(s) | The final follow-up data are entered, the data are sent to the doctor´s email and stored in the cloud. |  |  |  |
| Regular flow | - Doctor types in diagnose from the ICD 10 menue - Doctor types in Therapy (OPS-code from the OPS-code menue) - Doctor enters Dindo Clavien score - Doctor answers, if patient is still alive - Doctor enters data of death if answer was no - Doctor enters last date of follow up, if answer is yes - Doctor presses finish |  |  |  |
| Variants alternative flows, exceptions* | Only one workflow allowed. This is a clear A to B to C to D process that does not allow alternative ways. There will be a warning before pressing finish if data entered are correct as pressing finish removes the patient from the screen. | Please document if user found alternative way. | | |

Time required: _____minutes _____seconds

**Use Scenarios and ASQ**

**PATIENTS**

**Scenario P1:**

**You are the patient. In this task you will perform the onboarding on the system.**

**Please tell your supervisor when you are ready, they will start taking the time and put in the following data for your virtual patient until the end or follow the advices on the app.**

1. Starting point is the QR code
2. Install the app
3. Connect the smartwatch with your phone and login
4. At the end you should have launched the app and see the dashboard

**ASQ**

**Supervisor evaluation sheet with the expected walkthrough**:

### Scenario P1: Patient onboarding

| Core task | <UR13> Patient onboarding | Done with ease | Done with problems | Failed |
| --- | --- | --- | --- | --- |
| Short description | Patients have successfully downloaded the app and are guided though safe and successful connection with their smartwatch |  |  |  |
| Pre-condition(s) | The software is not connected to the smartwatch. |  |  |  |
| Post-condition(s) | The software is connected to the smartwatch. |  |  |  |
| Regular flow | - Patient activates smartwatch - Patient activates Bluetooth and connects accessory with smartphone - Patient follows set up advice on smartwatch |  |  |  |
| Variants alternative flows, exceptions* | Starting point may vary | Please document if user found alternative way. | | |

Time required: _____minutes _____seconds

**Scenario P2:**

**You are the patient. In this task you will explore the functions of the system without starting an exercise.**

**Please tell your supervisor when you are ready, they will start taking the time and put in the following data for your virtual patient until the end or follow the advice on the app.**

1. Starting point is the dashboard
2. Access all tab bar icons and return to the dashboard after each icon content has been explored

**ASQ**

**Supervisor evaluation sheet with the expected walkthrough**:

### Scenario P2: Patient exploration of App

| Core task | <UR14> Patient exploration of App | Done with ease | Done with problems | Failed |
| --- | --- | --- | --- | --- |
| Short description | Patients know that the dashboard is their overview, they know that the heart symbol leads them to the exercises, and they know that on the calendar they can look up individual exercises already performed and their schedule. |  |  |  |
| Pre-condition(s) | The patients are not trained in the dashboard, heart symbol and calendar use and views. |  |  |  |
| Post-condition(s) | The patients are trained in the dashboard, heart symbol and calendar use and view. |  |  |  |
| Regular flow | - Patient accesses dashboard - Patient moves to heart icon and is referred to exercise area - Patient accesses calendar and individual performed exercises or scheduled exercises. |  |  |  |
| Variants alternative flows, exceptions* | Starting point may vary. | Please document if user found alternative way. | | |

Time required: _____minutes _____seconds

**Scenario P3:**

**You are the patient. In this task you will launch a 6MWT and an exercise without performing it.**

**in the following data for your virtual patient until the end or follow the advice on the app.**

1. Starting point is the dashboard
2. Access all tab bar icons and return to the dashboard after each icon content has been explored

**ASQ**

**Supervisor evaluation sheet with the expected walkthrough**:

### Scenario P3: Patient knows how to start a 6MWT and exercise

| Core task | <UR15> Patient is familiar with launching exercise | Done with ease | Done with problems | Failed |
| --- | --- | --- | --- | --- |
| Short description | Patients know how to launch a 6 minute walking test or an exercise from their schedule |  |  |  |
| Pre-condition(s) | The patients open the dashboard and are supposed to start an exercise |  |  |  |
| Post-condition(s) | The patients finish the exercise. |  |  |  |
| Regular flow | - Patients start from the dashboard - Patients access heart icon - Patients choose either an exercise or the 6MWT - Patients wear their accessory and have it connected to the software - Patients follow the advice of the 6MWT/exercise - Patients finish the 6MWT/exercise and are prompted to the symptom check survey. |  |  |  |
| Variants alternative flows, exceptions* | Clear process from A to B. No variations allowed. | Please document if user found alternative way. | | |

Time required: _____minutes _____seconds

**Scenario P4:**

**You are the patient. In this task you will fill out the symptom check survey at the end of each exercise.**

**Please tell your supervisor when you are ready, they will start taking the time and put in the following data for your virtual patient until the end or follow the advice on the app.**

1. Starting point is the end of an exercise.
2. Fill out the symptom check survey and return to the dashboard.

**ASQ**

**Supervisor evaluation sheet with the expected walkthrough**:

### Scenario P4: Patient symptom checker survey

| Core task | <UR16> Patient symptom checker | Done with ease | Done with problems | Failed |
| --- | --- | --- | --- | --- |
| Short description | Patients are able to fill the survey out. |  |  |  |
| Pre-condition(s) | The symptom survey is empty. |  |  |  |
| Post-condition(s) | The symptom survey is filled out completely and the patient prompted back to the dashboard. |  |  |  |
| Regular flow | - Patients are automatically prompted to the symptom check survey - Patients fill out the survey and follow the warnings - Patients finish the survey by pressing finish and if necessary follow the plausibility check. |  |  |  |
| Variants alternative flows, exceptions* | Clear process from A to B. No variations allowed. | Please document if user found alternative way. | | |

Time required: _____minutes _____seconds

**Scenario P5:**

**You are the patient. In this task you will access and respond to the automated push notifications.**

**Please tell your supervisor when you are ready, they will start taking the time and put in the following data for your virtual patient until the end or follow the advices on the app.**

1. Starting point is the dashboard.
2. React to the notifications.

**ASQ**

**Supervisor evaluation sheet with the expected walkthrough**:

### Scenario P5: Patient notification and warnings

| Core task | <UR17> Patient symptom checker | Done with ease | Done with problems | Failed |
| --- | --- | --- | --- | --- |
| Short description | Patients react and notify to the automated push-notification system. |  |  |  |
| Pre-condition(s) | The push notifications are not confirmed or answered. |  |  |  |
| Post-condition(s) | The patient has read and confirmed the automated push notifications hat were displayed in the communication icon. |  |  |  |
| Regular flow | - Patients are on the dashboard and see notifications under the communication icon. - Patients access the icon and read every single push-message and confirm that they have read and understood the message. - Patients can return to the dashboard |  |  |  |
| Variants alternative flows, exceptions* | The sequence of responding to the notifications is not terminated. | Please document if user found alternative way. | | |

Time required: _____minutes _____seconds

**Scenario P6:**

**Misuse and Alarms**

**You are the patient. In this task you will react to alarms during an exercise. The exercise is set up that**

**alarms will be launched, and you need to react in an appropriate way to the alarms and warnings.**

**Please tell your supervisor when you are ready, they will start taking the time and put in the following**

**data for your virtual patient until the end or follow the advises on the app.**

1. Starting point is the dashboard.

2. Start and perform the exercise and follow the alarms and advice on the display.

**ASQ**

**Supervisor evaluation sheet with the expected walkthrough**:

**Scenario P6 Misuse and alarms**

| Core task | <UR-Alarms> Alarm response | Done with ease | Done with problems | Failed |
| --- | --- | --- | --- | --- |
| Short description | Patients react to alarms and haptic feedbacks and adapt their behavior accordingly. |  |  |  |
| Pre-condition(s) | Patients work-out in misuse areas. |  |  |  |
| Post-condition(s) | Patients return to adequate areas of action. |  |  |  |
| Regular flow | - Patients realize vibration alarms (ask them if sth changed) - Patients realize color change (ask them if sth changed) - Patients react to voice alarms - Patients adapt behavior as advised |  |  |  |
| Variants alternative flows, exceptions* | Clear process from A to B. No variations. | Please document if patient found alternative way. | | |

Time required: _____minutes _____seconds
